# Three hours of intermittent hypoxia increases circulating glucose levels in healthy adults

**DOI:** 10.14814/phy2.13106

**Published:** 2017-01-13

**Authors:** Lauren P. Newhouse, Michael J. Joyner, Timothy B. Curry, Marcello C. Laurenti, Chiara Dalla Man, Claudio Cobelli, Adrian Vella, Jacqueline K. Limberg

**Affiliations:** ^1^ Department of AnesthesiologyMayo ClinicRochesterMinnesota; ^2^ Department of Information EngineeringUniversity of PaduaPaduaItaly; ^3^ Department of EndocrinologyMayo ClinicRochesterMinnesota

**Keywords:** Hyperglycemia, insulin resistance, oral glucose tolerance test, sleep apnea

## Abstract

An independent association exists between sleep apnea and diabetes. Animal models suggest exposure to intermittent hypoxia, a consequence of sleep apnea, results in altered glucose metabolism and fasting hyperglycemia. However, it is unknown if acute exposure to intermittent hypoxia increases glucose concentrations in nondiabetic humans. We hypothesized plasma glucose would be increased from baseline following 3 h of intermittent hypoxia in healthy humans independent of any effect on insulin sensitivity. Eight (7M/1F, 21–34 years) healthy subjects completed two study visits randomized to 3 h of intermittent hypoxia or continuous normoxia, followed by an oral glucose tolerance test. Intermittent hypoxia consisted of 25 hypoxic events per hour where oxygen saturation (SpO_2_) was significantly reduced (Normoxia: 97 ± 1%, Hypoxia: 90 ± 2%, *P* < 0.01). Venous plasma glucose concentrations were measured on both visits before and after the 3 h protocol. No changes in plasma glucose were observed from baseline after 3 h of continuous normoxia (5.1 ± 0.2 vs. 5.1 ± 0.1 mmol/L, *P* > 0.05). In contrast, circulating glucose concentrations were increased after 3 h of intermittent hypoxia when compared to baseline (5.0 ± 0.2 vs. 5.3 ± 0.2 mmol/L, *P* = 0.01). There were no detectable changes in insulin sensitivity following intermittent hypoxia when compared to continuous normoxia, as assessed by the oral glucose tolerance test (*P* > 0.05). Circulating glucose is increased after 3 h of intermittent hypoxia in healthy humans, independent of any lasting changes in insulin sensitivity. These novel findings could explain, in part, the high prevalence of diabetes in patients with sleep apnea and warrant future studies to identify underlying mechanisms.

## Introduction

More than 18 million people in the United States have sleep apnea (Neubauer 2001) and up to 40% of these individuals will develop diabetes (Meslier et al. 2003). The association between sleep apnea and diabetes is independent of other comorbidities such as obesity, age, and sex (Meslier et al. 2003; Foster et al. 2009; Marshall et al. 2009; Dempsey et al. 2010; Pamidi et al. 2010; Rajan and Greenberg 2015). Treatment with continuous, positive airway pressure (CPAP) can improve glucose tolerance in patients with sleep apnea (Dorkova et al. 2008), further supporting a causative role of sleep apnea in impaired glucose homeostasis. Although the mechanisms underlying this association are unclear, recent studies of animal models indicate that intermittent hypoxia plays a key role in the metabolic dysfunction associated with sleep apnea. For example, mice exposed to intermittent hypoxia over a 4–6 week period exhibit fasting hyperglycemia (Shin et al. 2014). Additionally, rats exposed to short episodes (~1 h) of intermittent hypoxia also exhibit increased blood glucose concentrations (Rafacho et al. 2013). Although research in humans is limited, 30 min of continuous hypoxia results in glucose intolerance in healthy adults (Oltmanns et al. 2004). When hypoxia is administered intermittently for longer periods (8 h), impairments in glucose metabolism are observed [decreased insulin sensitivity, glucose effectiveness, and insulin secretion] (Louis and Punjabi 2009). The time course of this response – including whether a shorter exposure to intermittent hypoxia affects circulating glucose concentrations – has not been examined. Determining the effect of short‐duration intermittent hypoxia on plasma glucose levels is essential for understanding the initial mechanisms that contribute to the pathogenesis of hyperglycemia associated with sleep apnea, thereby identifying suitable therapeutic targets. We therefore sought to determine if 3 h of intermittent hypoxia would increase plasma glucose concentrations in healthy humans. We hypothesized plasma glucose levels would be increased from baseline in young healthy adults after 3 h of intermittent hypoxia, independent of any changes in insulin sensitivity.

## Methods

### Ethical approval

All procedures were reviewed and approved by the Institutional Review Board at Mayo Clinic. Informed consent was obtained in writing from all participants prior to the study.

### Subjects

Eight (7M/1F) healthy, nonobese (BMI <30 kg/m^2^) adults between the ages of 21–34 years completed this study. Exclusion criteria included a history of sleep apnea, diabetes, hepatic, renal, or hematological disease (e.g., peripheral vascular disease, stroke, hypertension), neurological disorders, bleeding/clotting disorders, and/or a history of substance abuse. Participants actively losing weight or taking medications known to affect glucose metabolism (e.g., corticosteroids, tricyclic‐antidepressants, benzodiazepines, opiates, barbiturates, anticoagulants) were also excluded. Participants refrained from alcohol, caffeine, and exercise for 24 h and fasted for 12 h prior to the study visit. Following informed consent, subjects met with a registered dietician to design a pretesting diet to ensure maintenance of body weight and controlled nutrition requirements prior to the study visits.

### Study days

Participants completed two study days in random order separated by a minimum of 1 week. During one study day, participants were exposed to normoxic conditions. On the other study day, participants were exposed to intermittent hypoxia (subjects were blinded to study condition). The female participant was studied during the placebo phase of oral contraceptive use. Her study visits were separated by 4 weeks and a negative urine pregnancy test was confirmed within 24 h of each study visit. On the evening prior to each study day, participants reported to the Clinical Research and Trials Unit at the Mayo Clinic at 17.00 h, consumed a standard 10 kcal/kg meal (55% carbohydrates, 30% fat, 15% protein) between 18.00 h and 18.30 h (followed by a 12‐h fast), and slept for a standard 8–9 h [with oxygen saturation and heart rate monitored continuously during sleep by portable pulse oximetry (Nonin 3150 WristOx2 Wrist‐Worn Pulse Oximeter, Minneapolis, MN)].

### Instrumentation

On the morning of the study, participants awoke at 06.15 h and a retrograde intravenous catheter was placed in the dorsum of the non‐dominant hand under local anesthesia (2% lidocaine) for periodic blood sampling. Participants wore a mask connected to a two‐way non‐rebreathing valve for administration of gasses and to allow for continuous measurement of tidal volume, breathing frequency (Universal Ventilation Meter, Ventura, CA), and inspired/expired gasses (Cardiocap/5, Datex‐Ohmeda, Louisville, CO).

### Protocol

Following instrumentation, all participants were exposed to 3 h of either intermittent hypoxia or continuous normoxia. During the 3‐h protocol, venous blood samples were drawn at 30 min intervals for measurement of glucose and insulin. Participants then completed a 2‐h, frequently sampled oral glucose tolerance test. During all tests, participants rested quietly, semisupine in a hospital bed and were allowed to watch movies and/or listen to music. Sleeping was not permitted (Louis and Punjabi 2009).

### Intermittent hypoxia

A two‐way switching valve was connected to the participant's mask and was used to administer acute intermittent hypoxia or continuous normoxia. Throughout the 3‐h protocol, a switching valve alternated between two gas reservoirs (meteorological balloons), resulting in 25‐second exposures to one balloon, followed by 2‐min exposure to the other balloon (Louis and Punjabi 2009). On the intermittent hypoxia day, one balloon was filled with hypoxic air (5% oxygen, 3% carbon dioxide, balance nitrogen) and the other with normoxic medical gas – resulting in 25 events per hour in which oxygen saturation was significantly reduced (normoxia: 97 ± 1%, hypoxia: 90 ± 2%, *P* < 0.01). On control days, both balloons were filled with normoxic medical gas and no changes in oxygen saturation were observed (normoxia: 98 ± 1%, normoxia: 97 ± 1%, *P* > 0.05).

### Oral glucose tolerance test

Following the 3‐h protocol, participants placed their nondominant hand with a retro‐grade intravenous catheter into a heated plexiglass box maintained at 55°C for collection of “arterialized” venous blood (Cobelli et al. 2014). After 15 min, a blood sample was taken (oral glucose tolerance test [OGTT] T_0_). Participants ingested a glucose drink (75 g glucose) over a period of 5 min. Arterialized, venous blood was then sampled periodically for 2‐h (T_10_, T_20_, T_30_, T_60_, T_90_, T_120_) for measurement of insulin, glucose, and C‐peptide (Cobelli et al. 2014).

### Blood samples

All blood samples were immediately placed on ice and centrifuged at 4°C after which the plasma was removed and stored at −80°C until analysis (Wehrwein et al. 2010). Samples were analyzed by Mayo Immunochemical Core Laboratory. Glucose was measured on the Roche Cobas c311 (Roche Diagnostics, Indianapolis, IN) utilizing a hexokinase reagent (Analytical measurement range: 2.2–41.7 mmol/L; Intra‐assay CV 0.6% at 4.6 mmol/L and 0.5% at 6.9 mmol/L; Inter‐assay CV 1.1% at 4.5 mmol/L and 1.1% at 6.8 mmol/L). Insulin was assessed using a two‐site immunoenzymatic assay performed on the DxI 800 automated immunoassay system (Beckman Instruments, Chaska, MN; Analytical measurement range: 0.7–2083 pmol/L; Intra‐assay CV 4.6% at 76.5 pmol/L and 2.5% at 489.9 pmol/L; Inter‐assay CV 6.2% at 36.8 pmol/L and 7.7% at 836.2 pmol/L) (Wehrwein et al. 2010). C‐peptide was measured by two‐site immunoenzymatic sandwich assay on the Roche Cobas e411 (Roche Diagnostics, Indianapolis, IN; Analytical measurement range: 0.1–13.3 nmol/L; Intra‐assay CV 2.8% at 1.6 nmol/L and 1.5% at 3.3 nmol/L; Inter‐assay CV 5.4% at 1.5 nmol/L and 5.4% at 4.4 nmol/L).

The oral minimal model utilized glucose and insulin concentrations during the OGTT to measure net insulin action (S_*i*_), as previously described (Van Cauter et al. 1992; Breda et al. 2001; Dalla Man et al. 2004). Similarly, glucose and C‐peptide concentrations were used to calculate *β*‐cell responsivity (Φ) to glucose, after accounting for age‐associated changes in C‐peptide clearance. The model assumes that insulin secretion is comprised of a static and dynamic component. The dynamic component is proportional to the rate of increase in glucose concentrations through the parameter *ϕ*_Dynamic_, defined as the dynamic responsivity index. The static component represents the provision of new insulin to the releasable pool and is proportional, through the parameter *ϕ*_Static_, to sustained elevations in glucose concentration. An index of total *β*‐cell responsivity to glucose (Φ) is derived from both indices. The disposition index (DI) was calculated as the product of S_*i*_ and Φ.

### Statistical analysis

Characteristics (e.g., height, weight) collected at baseline as well as responses to the OGTT were compared between visits (Normoxia, Intermittent Hypoxia) via a paired two‐tailed t‐test. The primary analysis examined the effect of 3 h of intermittent hypoxia or continuous normoxia on plasma glucose levels. To do this, plasma glucose levels were compared before (T_0_) and after (T_180_) the 3 h protocol within visits (one‐way repeated measures analysis of variance). The secondary analysis examined the time course of the glucose response. The main effect of time and condition (normoxia, intermittent hypoxia), as well as the interaction between time and condition, on main outcome variables were assessed using a two‐way repeated measures analysis of variance. Data are reported as the mean ± standard error and *P* ≤ 0.05 was considered statistically significant.

## Results

All subjects (7M, 1F) were young (28 ± 2 years), nonobese (height 178 ± 3 cm, weight 78 ± 6 kg, BMI 25 ± 1 kg/m^2^), nondiabetic (fasting glucose 5.1 ± 0.2 mmol/L; fasting insulin 26 ± 6 pmol/L; HOMA‐IR 1.0 ± 0.3), without sleep apnea (apnea hypopnea index <5 events/hour). No differences in baseline characteristics were observed between normoxia and intermittent hypoxia visits (*P*‐value range 0.13–0.79).

### Glucose and insulin concentrations during normoxia and intermittent hypoxia

**Figure 1:**
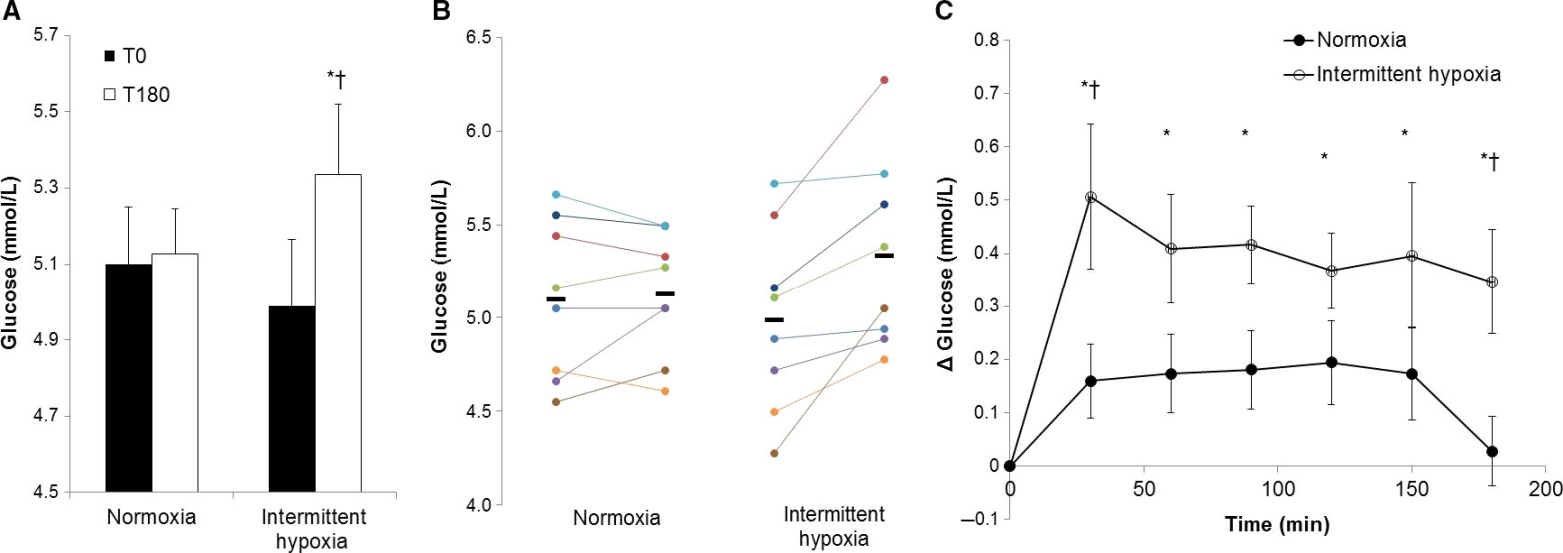
Plasma glucose following 3‐h of intermittent hypoxia or continuous normoxia. Data are reported as Mean±SEM from *n* = 8 (7M/1F). * *P* < 0.05 versus T_0_. † *P* ≤ 0.05 versus Control. A (average data) & B (individual data)**.** Glucose levels were increased by T_180_ on the intermittent hypoxia visit (*P < 0.01), but no change was observed on the normoxia visit (*P* = 0.75). There were no differences in glucose levels between visits at T_0_ (*P* = 0.27), but there was a difference at T_180_ (†*P* = 0.051). (C) Glucose levels remained unchanged during the control visit (*P* > 0.05 for all). During the intermittent hypoxia visit, glucose levels were greater than T_0_ at all time points (*P < 0.01 for all). When data were compared between visits, there was a significant difference at T_30_ (†*P* = 0.02) and T_180_ (†*P* = 0.03).

Data are reported in Table 1 and Figure**** 1. No changes in plasma glucose were observed after 3 h of continuous normoxia (*P* = 0.75). In contrast, circulating glucose concentrations were increased from baseline after 3 h of intermittent hypoxia (*P* < 0.01, Fig. 1A and B). When data were assessed *during* the 3 h protocol, circulating glucose was significantly augmented after 30 min of intermittent hypoxic exposure and remained elevated from baseline throughout the 3 h protocol; no changes in glucose were observed on the normoxic visit (interaction of time and condition, *P* = 0.045; Fig.**** 1C). No changes in circulating insulin levels were observed from baseline during 3 h of either continuous normoxia or intermittent hypoxia (main effect of time: *P* = 0.91; Table 1).

**Table 1:** Plasma glucose and insulin during 3 h of intermittent hypoxia or continuous normoxia

	T_0_	T_30_	T_60_	T_90_	T_120_	T_150_	T_180_	*2‐way Repeated Measures ANOVA*		
								*P‐*value (Time)	*P‐*value (Condition)	*P‐*value (Interaction)
Glucose (mmol/L)										
Normoxia	5.1 ± 0.2	5.3 ± 0.1	5.3 ± 0.1	5.3 ± 0.1	5.3 ± 0.2	5.3 ± 0.1	5.1 ± 0.1	**<0.01**	0.38	**0.045**
Intermittent hypoxia	5.0 ± 0.2	5.5 ± 0.2^a^	5.4 ± 0.2^a^	5.4 ± 0.2^a^	5.4 ± 0.2^a^	5.4 ± 0.2^a^	5.3 ± 0.2^a^			
Insulin (pmol/L)										
Normoxia	26 ± 6	30 ± 6	26 ± 4	25 ± 3	26 ± 4	26 ± 5	27 ± 5	0.91	0.88	0.42
Intermittent hypoxia	24 ± 4	25 ± 4	28 ± 5	25 ± 3	25 ± 5	29 ± 4	25 ± 4			

Data are reported as Mean ± SEM from *n* = 8 (7M/1F). Bold indicates significant values.

a *P* < 0.05 versus T_0_.

### Oral glucose tolerance test

**Figure 2:**
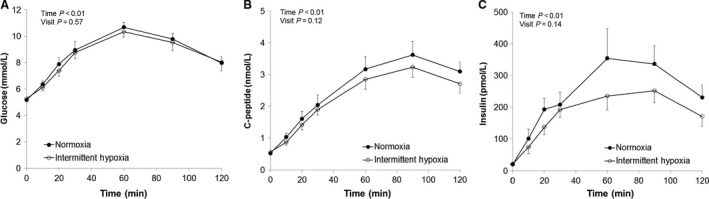
Oral glucose tolerance test. Data are reported as Mean±SEM from *n* = 8 (7M/1F). Any differences in the glucose (A, *P* = 0.57), C‐peptide (B, *P* = 0.12) or insulin (C, *P* = 0.42) responses to the oral glucose tolerance test (OGTT) were not detected following continuous normoxia versus intermittent hypoxia.

**Figure 3:**
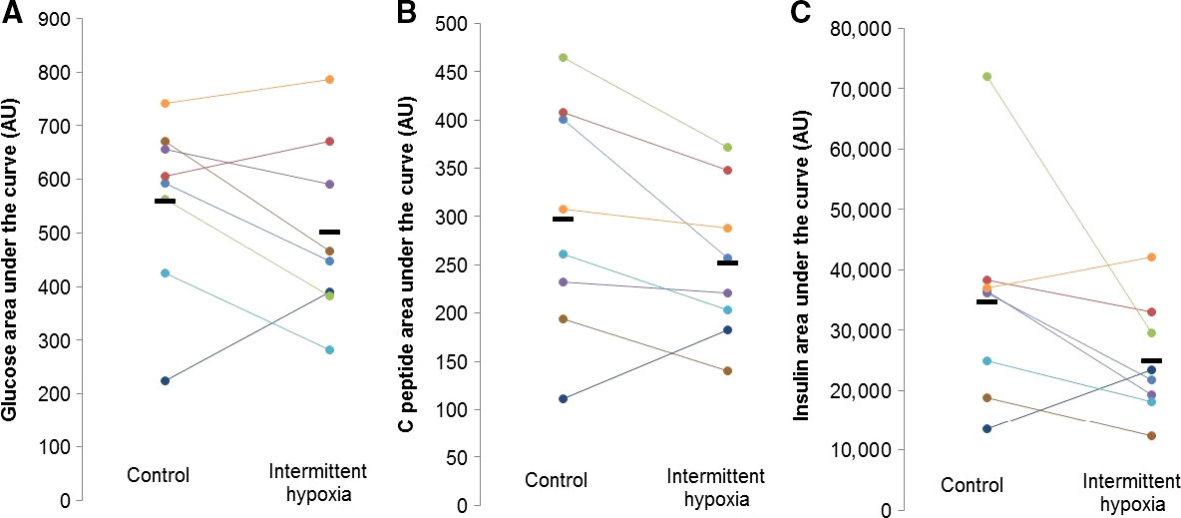
Oral glucose tolerance test – Area Under the Curve. Data are reported as Individual Responses from *n* = 8 (7M/1F). Any differences in the area under the curve responses for glucose (A**,** *P* = 0.27), C‐peptide (B, *P* = 0.08), or insulin (C, *P* = 0.13), to the oral glucose tolerance test (OGTT) were not detected following continuous normoxia versus intermittent hypoxia.

**Figure 4:**
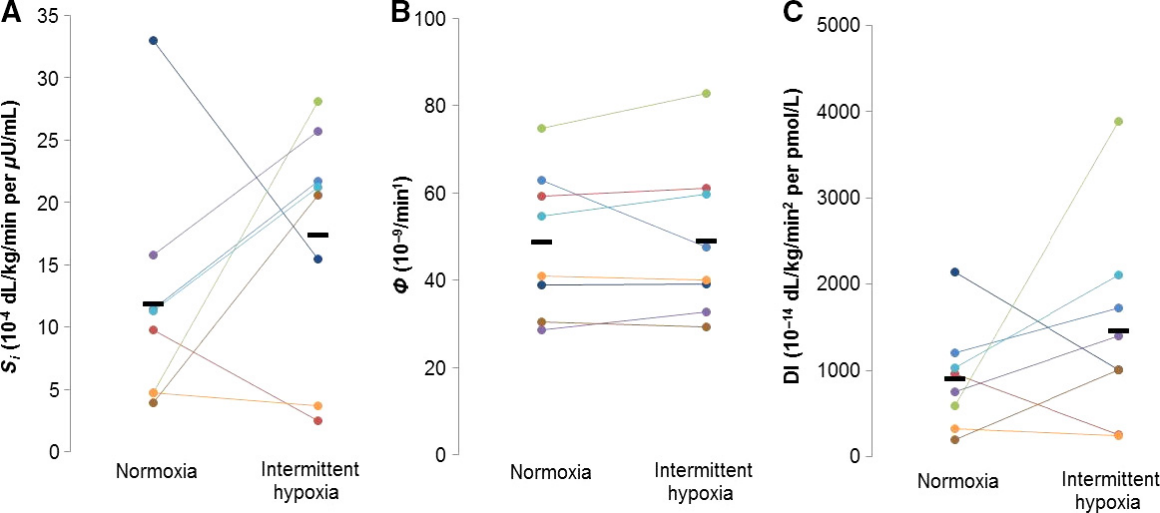
Oral glucose tolerance test. Data are reported as Individual Responses from *n* = 8 (7M/1F). Any differences in net insulin action (A: S_*i*_, *P* = 0.28), *β*‐cell responsivity (B: Φ, *P* = 0.93), and disposition index (C: DI,* P* = 0.28) were not detected following continuous normoxia versus intermittent hypoxia.

In response to the OGTT, significant increases were observed in glucose, insulin, and C‐peptide over time (main effect of time, *P* < 0.01; Fig.**** 2). The increase in glucose, insulin, and C‐peptide during the OGTT were not significantly different between visits (main effect of condition, *P*‐value range 0.12–0.57; Fig.**** 2); similar conclusions were made when assessed as an area under the curve (*P*‐value range 0.08–0.27; Fig.**** 3). Furthermore, net insulin action (S_*i*_: 12 ± 3 vs. 17 ± 3 10^−4^ dL/kg/min per *μ*U/mL, *P* = 0.28), *β*‐cell responsivity (Φ: 49 ± 6 vs. 49 ± 6 10^−9^/min, *P* = 0.93; *ϕ*_Dynamic_: 629 ± 64 vs. 662 ± 52 10^−9^, *P* = 0.41; *ϕ*_Static_: 43 ± 6 vs. 42 ± 6 10^−9^/min, *P* = 0.46), and disposition index (DI: 901 ± 216 vs. 1453 ± 417 10^−14^ dL/kg/min^2^ per pmol/L, *P* = 0.28) were not different following continuous normoxia and intermittent hypoxia, respectively (See Fig.**** 4).

## Discussion

These data are the first in humans to show immediate (30 min) and prolonged (up to 180 min) increases in circulating plasma glucose levels during acute exposure to intermittent hypoxia. Our findings are consistent with prior work from animal (Rafacho et al. 2013) and human models (Louis and Punjabi 2009); however, they suggest shorter (<8 h) exposures of intermittent hypoxia can elicit very early changes in circulating glucose levels. Furthermore, these are the first data to show initial increases in plasma glucose in response to intermittent hypoxia occur independent of changes in insulin sensitivity. With this, observed increases in glucose with acute exposure to intermittent hypoxia in humans may be attributed to many potential mechanisms. We will briefly discuss some possibilities below, as they relate to present findings.

First, insulin sensitivity may be altered in response to intermittent hypoxia. Data from animal models have shown that 30 days of intermittent hypoxia may impair *β*‐cell function resulting in augmented basal insulin secretion, insulin resistance, defective proinsulin processing, and impaired glucose‐stimulated insulin secretion (Wang et al. 2013). Previous data following an 8 h exposure to intermittent hypoxia in healthy humans have also shown worsening insulin sensitivity and glucose effectiveness (Louis and Punjabi 2009). This idea is supported clinically, given insulin resistance in patients with chronic obstructive pulmonary disease can be reversed with acute supplemental oxygen (Jakobsson and Jorfeldt 2006) and patients with sleep apnea and metabolic syndrome exhibit insulin resistance that improves with CPAP therapy (Dorkova et al. 2008). In contrast to longer exposures, we did *not* observe any changes in insulin sensitivity (S_*i*_) following 3 h of intermittent hypoxia (Fig.**** 4). Despite no changes in insulin sensitivity, we observed an increase in circulating glucose concentrations (Table 1 **,** Fig.**** 1). Together, our data suggest intermittent hypoxia produces hyperglycemia independent of changes in insulin resistance, at least initially.

Despite limited effects on insulin secretion and/or sensitivity, our data suggest that hepatic insulin extraction may be augmented following intermittent hypoxia exposure. Consistent with previous work in humans showing preserved insulin secretion following intermittent hypoxia (Louis and Punjabi 2009), our data suggest the portal vein concentrations of insulin are unchanged (as identified by relatively preserved C‐peptide levels during an OGTT following intermittent hypoxia, Fig.**** 2). However, it appears the amount of insulin the liver allows into the systemic circulation may be attenuated by intermittent hypoxia (Fig.**** 2, trend for reduced insulin response to OGTT, *P* = 0.14). These data are consistent with previous work suggesting hypoxic exposure has little effect on insulin sensitivity and glucose tolerance, but may exert its effect on insulin clearance (Simpson et al. 2016).

It is also reasonable to propose these early changes in plasma glucose in response to intermittent hypoxia may be the result of an increase in adipose tissue lipolysis; however, work in humans is limited. A strong body of literature from animal models supports the idea that lipolysis is increased in response to chronic intermittent hypoxia (Drager et al. 2010). Consistent with this, free fatty acid levels are increased in patients with sleep apnea and are associated with higher apnea hypopnea indices (Barceló et al. 2011). Furthermore, free fatty acid levels increase during sleep in patients with sleep apnea, and this increase can be prevented by supplemental oxygen (Jun et al. 2011). However, in one study in humans, 6 h of intermittent hypoxia increased plasma glucose levels but measures of lipolysis were unaffected (Mahat et al. 2016). Combined with limited effects on insulin secretion and/or sensitivity in the present investigation, these data suggest lipolysis may be a longer term consequence of intermittent hypoxia.

Over the last ~10 years, the carotid bodies have been shown to play an important role in glucose sensing/regulation in both in vitro (Pardal and Lopez‐Barneo 2002) and in vivo (Wehrwein et al. 2010) models. Shin and colleagues (2014) found that mice exposed to intermittent hypoxia exhibited fasting hyperglycemia that was attenuated via carotid body denervation (Shin et al. 2014). The authors proposed this response was the result of increased sympathetic activation leading to increases in hepatic glucose output (Shin et al. 2014). Consistent with this idea, other groups suggest that intermittent hypoxia alters glucose output from the liver via a sympathetically mediated mechanism (Trzepizur et al. 2015). Along these lines, glucose infusion rates during a hyperinsulinemic euglycemic clamp in humans are lower during hypoxia and can be attenuated with sympathetic inhibition (transdermal clonidine) (Peltonen et al. 2012). Acute exposures (<30 min) to intermittent hypoxia are known to increase sympathetic outflow (Morgan 2007). Sympathetically mediated vasoconstriction of the skeletal muscle vasculature could also contribute to a reduction in glucose unloading, therefore increasing circulating glucose levels. Thus, it is likely the early (~30 min, Fig.**** 1C) changes in plasma glucose levels may be sympathetically mediated (Morgan 2007), either as a result of decreases in glucose unloading (Khoury and McGill 2011; Peltonen et al. 2012), a reduction in glucose‐stimulated insulin secretion (Jun et al. 2014), or increased endogenous glucose production (Yi et al. 2010; Shin et al. 2014). Further mechanistic studies are warranted.

### Experimental considerations

This is the first study of its kind to show immediate increases in plasma glucose with exposure to intermittent hypoxia that are not due to insulin resistance. However, it is important to acknowledge the OGTT was performed under normoxic conditions. This assumes that any effect of intermittent hypoxia is relatively prolonged. Importantly, when the first glucose sample was taken for the OGTT (~20 min following exposure to intermittent hypoxia, T_0_), glucose remained above baseline on the intermittent hypoxia study day (5.0 ± 0.2 vs. 5.3 ± 0.3 mmol/L; *P* < 0.01), but no differences were found on the control day (5.1 ± 0.2 vs. 5.2 ± 0.1 mmol/L; *P* = 0.49). Therefore, it appears the effect of intermittent hypoxia on altered glucose regulation is prolonged and does not support the idea of a “rapid onset, rapid offset” phenomenon. To further explore this idea, future studies should consider: (1) measuring glucose for 1–2 h following intermittent hypoxia exposure, (2) conducting an OGTT during a short exposure (<3 h) of intermittent hypoxia to confirm present findings collected during normoxia. Further studies should also consider measuring endogenous glucose production, to better determine the source of the increased glucose. It is also important to note that the degree of SpO_2_ desaturation reached by participants in this study was less than the desaturation typical in patients with sleep apnea (Dempsey et al. 2010). Additionally, the degree of desaturation in our study was also much less than the typical level of desaturation observed in the animal protocols cited (Shin et al. 2014; Rafacho et al. 2013). It is likely that a greater desaturation would result in a more prominent surge in glucose. Furthermore, the IH exposure experienced by patients with sleep apnea often occurs at irregular intervals, which is in contrast to the regular timing of IH exposure in our protocol. The irregularity of the timing of desaturation observed in sleep apnea is likely an important covariate.

### Clinical implications

Present studies were conducted in healthy, lean, recreationally active individuals with no history of sleep apnea and/or diabetes. For this reason, it would be interesting to repeat these studies in more at‐risk populations, including individuals that are insulin resistant. We propose that the effects we observed would be exacerbated in such individuals and could shed light on the likely complicated role of insulin in the initial changes in circulating glucose with intermittent hypoxia. To translate these findings to clinical sleep apnea, future work should also consider longer, repeated (multiple days) exposures to intermittent hypoxia in addition to similar protocols conducted at night and/or during sleep. Lastly, it would be of clinical interest to look at these parameters after the participant is both sedentary and active; physical activity is known to increase glucose utilization and could attenuate any observed increases in plasma glucose. Such experiments could thus provide important mechanistic insights into potential therapies for at‐risk individuals.

## Conclusions

The present novel findings are the first in humans to show plasma glucose levels are increased after 30 min of intermittent hypoxia and this increase is maintained throughout 3 h of intermittent hypoxia (and for at least 20 min post). Contrary to previous studies in humans over longer time periods, our analyses reveal that the altered glucose handling after 3 h of intermittent hypoxia is unlikely to be the result of changes in insulin sensitivity/resistance. This suggests the initial mechanisms behind the increase in plasma glucose in response to intermittent hypoxia differ from more long‐term exposure and likely play an important role in the progression of the response. Further investigations will be needed to determine the role for specific mechanisms (e.g., changes in peripheral blood flow and glucose distribution, as well as changes in gluconeogenesis and lipolysis) in the elevated glucose levels observed following acute intermittent hypoxia in humans. Given the high prevalence of diabetes in sleep apnea, these data may have important implications in altered glucose handling observed in patients with sleep apnea. By understanding the initial mechanisms behind this association, future studies will better be able to understand important pathways in the pathogenesis of these diseases. Importantly, our data have furthered our understanding of the relationship between two prevalent human diseases and set the stage for further clinical research that will benefit to a very large patient demographic.

## Conflict of Interest

None declared.
